# Supplementary material for: Four-dimensional left ventricular motion clustering reveals cardiovascular phenotypes at population scale
Source: Sci Rep. 2026 Jun 11;16:26725. doi: 10.1038/s41598-026-56151-y (PMC13507085; doi:10.1038/s41598-026-56151-y)
Supplement: Supplementary file 1 [file 41598_2026_56151_MOESM1_ESM.pdf]

Supplementary Material

Supplementary Figures and Tables

Mass univariate analysis of signature geometry differences

Temporal signatures extracted from the latent space of the convolutional variational autoencoder were stratified into six distinct phenogroups using time series clustering. To assess localised differences in the trajectory geometry of signatures across clusters, we used a mass univariate analysis using permuted ordinary least squares (OLS) regression, as implemented in the Python module `nilearn`[1]. This method tests each feature, in this case each time point along the trajectory, independently for group-level differences, while accounting for the dependency structure through non-parametric permutation testing. This allows to avoid strong assumptions about the distribution of residuals. Formally, for each time point t, a linear model of the form

$$y_t = X\beta_t + \epsilon_t$$

was fitted, where y_t is the temporal signature value at time t, X is the matrix encoding phenogroup attribution, β_t is the coefficient vector, and ϵ_t is the residual error. The significance of the coefficients was assessed through $n = 10{,}000$ permutations of the group labels, giving empirical null distributions and corrected P-values.

This analysis was performed for each pairwise combination of phenogroups and separately for the trajectories projected onto the first two principal components (PC1 and PC2), which captured the dominant axes of temporal variation. All resulting P-values were corrected for multiple comparisons using the Benjamini-Hochberg procedure, with a significance level threshold of $P < 0.05$.

As shown in Supplementary Figure 7, we observed statistically significant differences at all trajectory time points for every pair of phenogroups. However, the specific components (PC1 vs. PC2) showing differences varied across comparisons. For example, PG1 was most similar to PG3, consistent with their overlapping average trajectory shapes. Conversely, PG4 exhibited marked dissimilarity from all other phenogroups, particularly PG6, while PG2 and PG5 shared a relatively larger number of similar trajectory coordinates. Interestingly, PG6 differed substantially at the individual level from PG5, despite a similar average trajectory. This could reflect greater intra-cluster heterogeneity driven by the inclusion of extreme phenotypes: PG5 represented high-risk individuals with clinical events, while PG6 gathered an even higher-risk subgroup, potentially introducing more variation in latent temporal features.

Number of clusters	2	**3**	4	5	6	7
Fold 1	0.949	0.971	0.513	0.656	0.599	0.447
Fold 2	0.982	0.822	0.651	0.601	0.477	0.511
Fold 3	0.875	0.940	0.733	0.819	0.530	0.549
Fold 4	0.666	0.968	0.895	0.645	0.663	0.307
Fold 5	0.863	0.869	0.411	0.861	0.474	0.530
Average	0.867	**0.914**	0.641	0.716	0.549	0.469

Supplementary Table 1. Average Rand Index score for clustering on in-plane position. Stability of the clustering method based on in-plane position was assessed with an average Rand Index score for multiple chosen values of clusters and 5 partitions of equal size of the initial dataset.

Number of clusters	**2**	3	4	5	6	7
Fold 1	0.937	0.837	0.748	0.770	0.402	0.357
Fold 2	0.839	0.625	0.458	0.852	0.375	0.474
Fold 3	0.872	0.400	0.691	0.683	0.447	0.385
Fold 4	0.940	0.593	0.837	0.735	0.612	0.420
Fold 5	0.932	0.707	0.296	0.556	0.309	0.388
Average	**0.904**	0.632	0.606	0.719	0.429	0.405

Supplementary Table 2. Average Rand Index score for clustering on contour characteristics. The stability of the clustering method based on contour characteristics was assessed with an average Rand Index score for multiple chosen values of clusters and 5 partitions of equal size of the initial dataset.

Subset	In-plane position ARI	Contour ARI
Subset 1	0.950	0.373
Subset 2	0.932	0.791
Subset 3	0.819	0.933
Subset 4	0.946	0.398
Subset 5	0.920	0.435
Average	**0.913**	**0.586**

Supplementary Table 3. Adjusted Rand Index score for PCA stability analysis. The sensitivity of the phenogroup assignments to the PCA decomposition was assessed by recomputing PCA on five stratified 80% subsets of the cohort (16,281 patients each) and comparing the resulting cluster labels to those of the full-dataset pipeline using the adjusted Rand index. Subset principal components were sign-aligned to the full-dataset components prior to clustering, and K-means was run with $n_{\text{init}} = 5$.

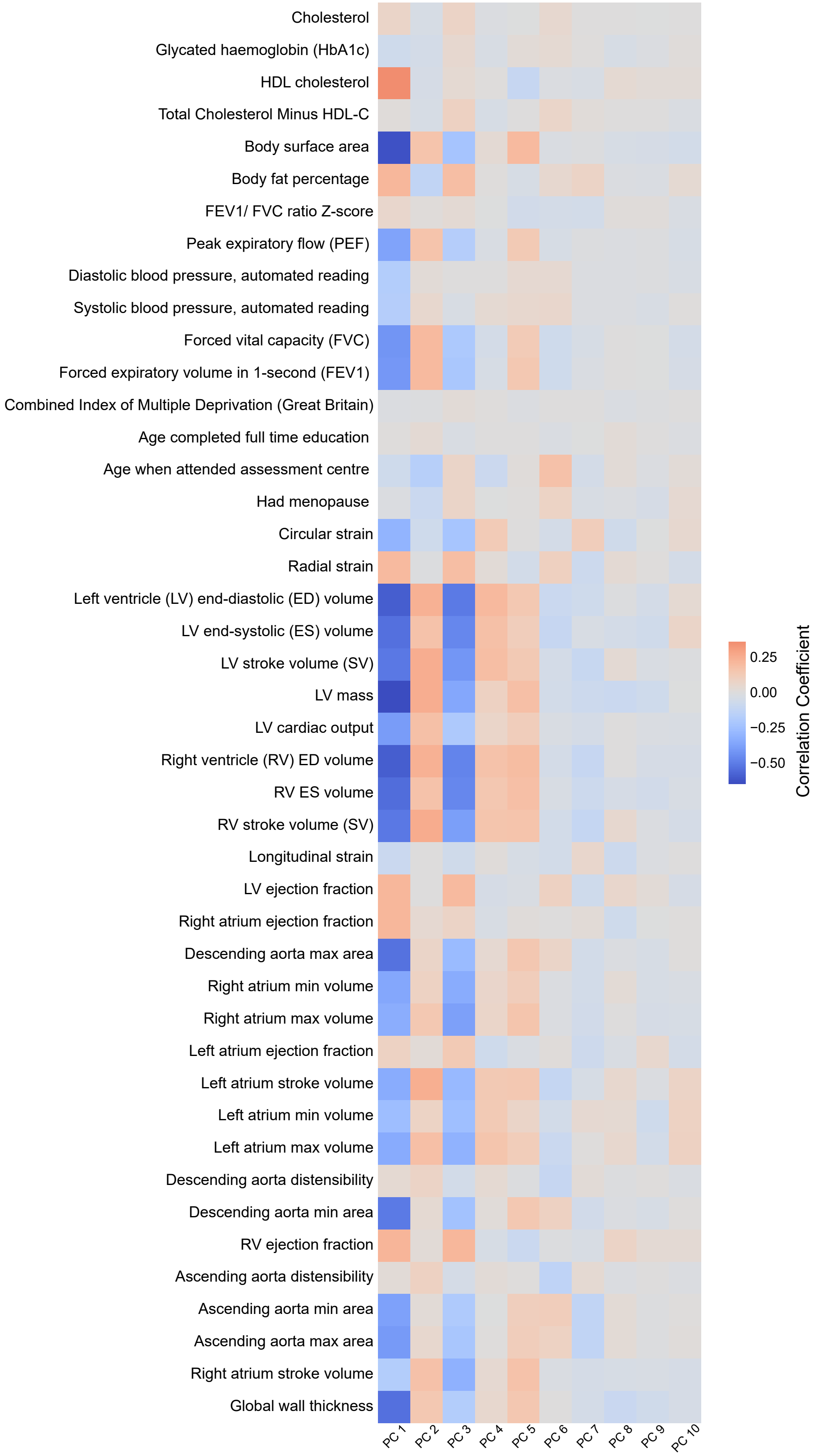

Supplementary Figure 1. Correlation between latent principal components and clinical features. A correlation analysis was performed between the first ten principal components of the latent space and relevant subject feature categories: demographics, body and clinical measurements, biomarkers, and cardiac MRI-derived measures.

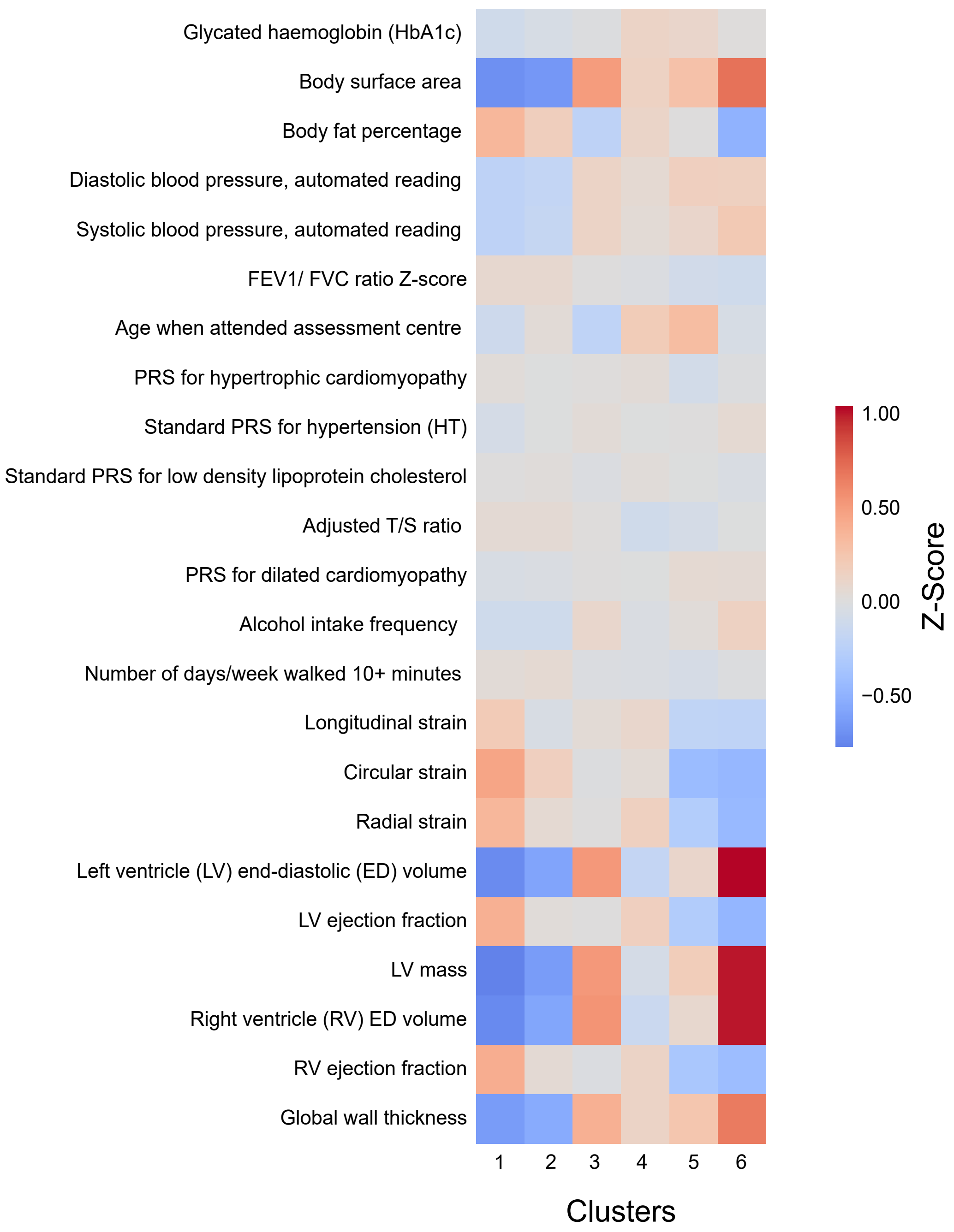

Supplementary Figure 2. Heatmap of z-scores of important features against clusters. Z-scores of each relevant feature are plotted for each of the six clusters. HbA1c, hemoglobin A1C; FEV1/FVC, ratio of the forced expiratory volume in 1-second to the forced vital capacity; T/S, telomere to single copy gene; PRS, polygenic risk score

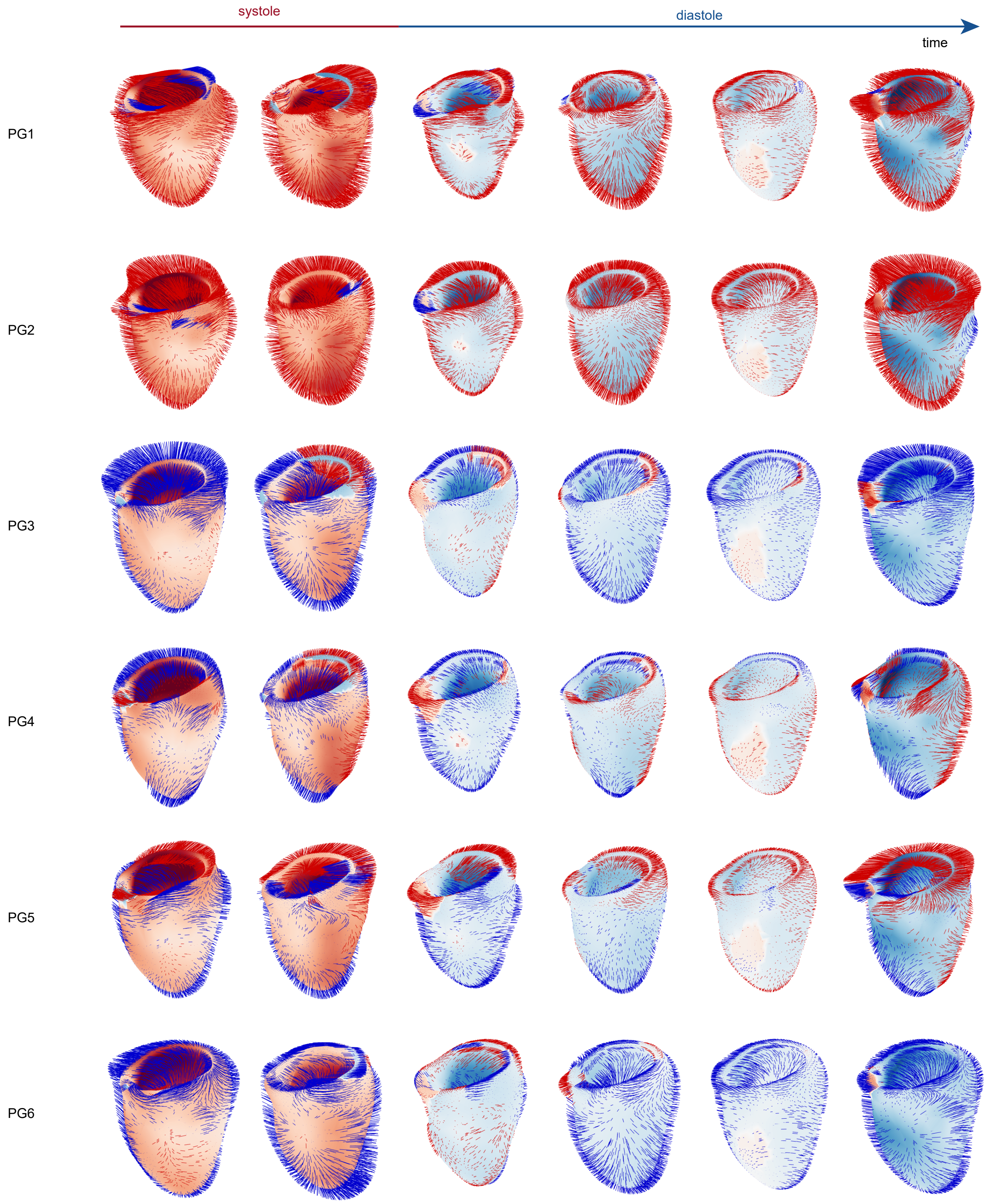

Supplementary Figure 3. Left ventricle motion velocity by phenogroup - Anteroseptal view. The left ventricle motion was compared to the population average motion for each phenogroup. The six frames represent one entire cardiac cycle from systole (contraction of the ventricle) to diastole (relaxation of the ventricle). The velocity of the ventricle was calculated as the local gradient of change between two consecutive frames, averaged out over multiple frames to reduce the number of displayed frames, and normalised for the size of the ventricle. Red beams indicate areas of faster movement relative to the population average motion, while blue beams show areas of relative slower movement.

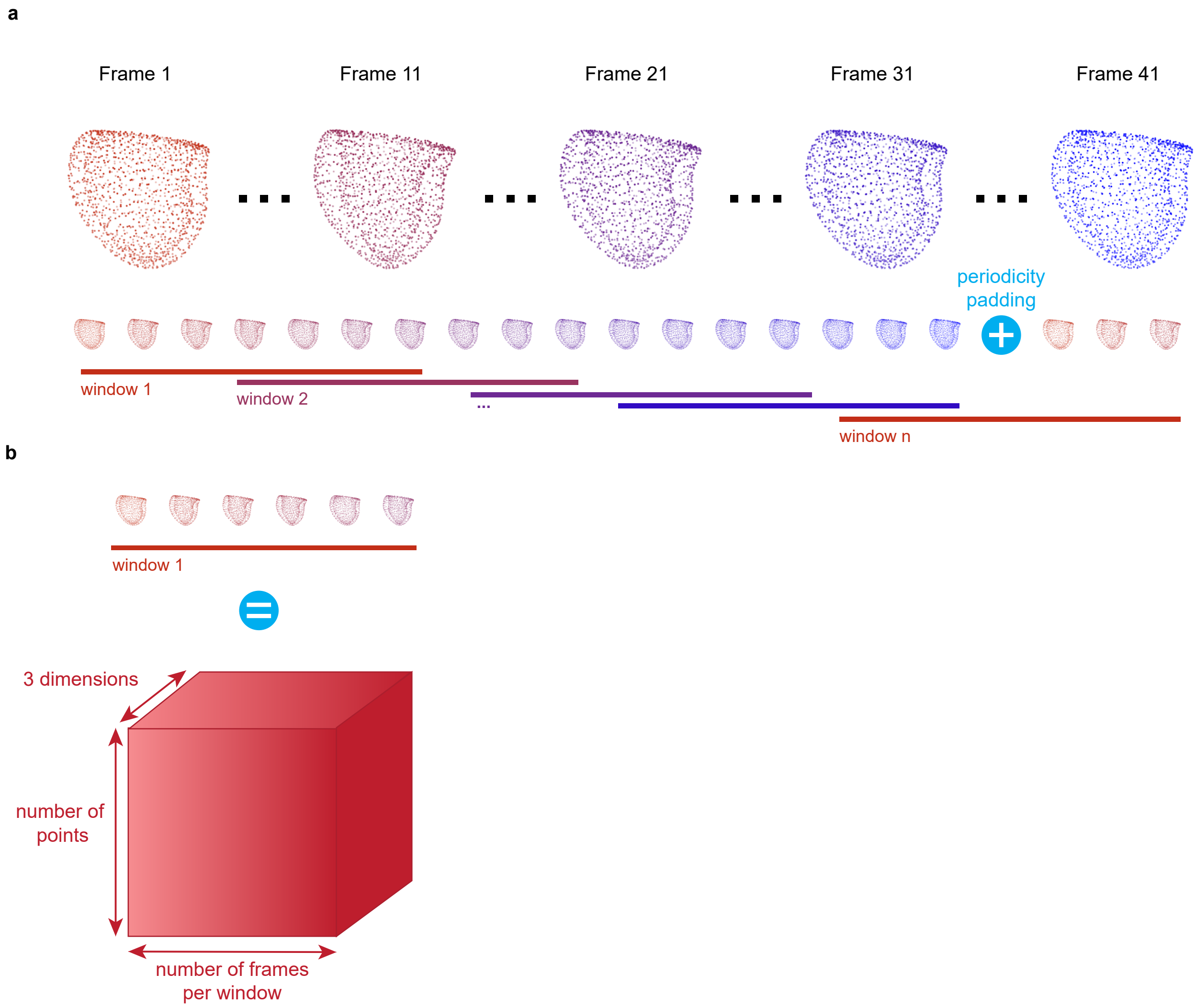

Supplementary Figure 4. Windows of cardiac motion. a. Windows of cardiac motion were created with a sliding window technique to embed the motion of the cardiac cycle in a single object. Periodicity padding was used to ensure that windows covered the entire cycle. **b.** Time windows were equivalent to a three-dimensional matrix in the mathematical space.

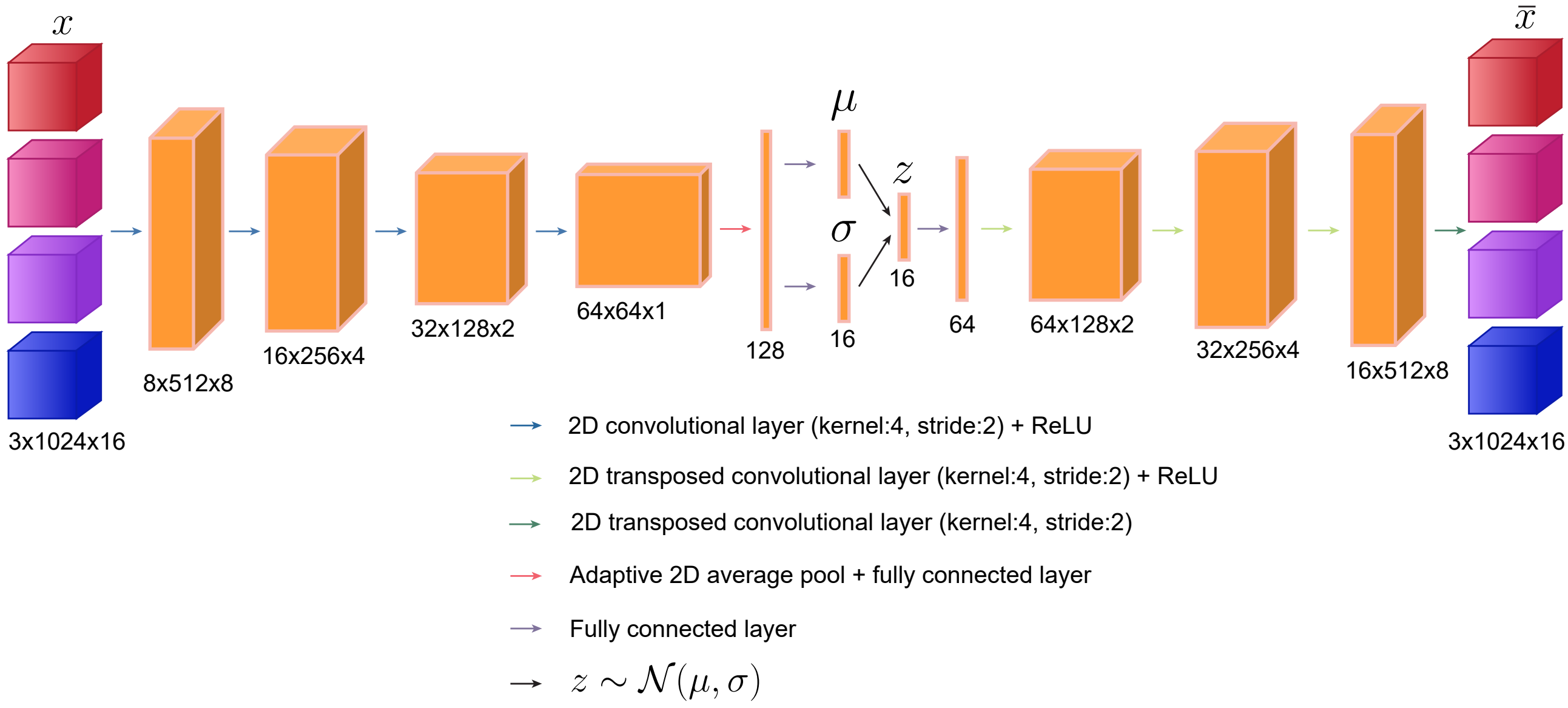

Supplementary Figure 5. Architecture of convolutional variational autoencoder. The convolutional autoencoder took the time windows x as input. Its encoder was made of four 2D convolutional layers all followed by a reLU (rectified linear unit) activation function. Before the bottleneck, adaptive 2D average pooling was applied to the output of the convolutional layers. The latent vector z was sampled from the latent distribution of mean μ and standard deviation σ. The latent vector was fed into the decoder with four 2D transposed convolutional layers, all followed by a reLU activation function except for the last layer. The output of the model was the reconstructed time windows $\bar{x}$.

Image-derived measure	Unit
Longitudinal strain	%
Circumferential strain	%
Radial strain	%
LV end-diastolic volume	mL
LV end-systolic volume	mL
LV stroke volume	mL per beat
LV cardiac output	mL per minute
LV ejection fraction	%
LV mass	mg
Global wall thickness	mm
RV end-diastolic volume	mL
LV end-systolic volume	mL
RV stroke volume	mL per beat
RV ejection fraction	%
Ascending aorta max area	mm2
Ascending aorta min area	mm2
Ascending aorta distensibility	10^3 mmHg^{-1}
Descending aorta max area	mm2
Descending aorta min area	mm2
Descending aorta distensibility	10^3 mmHg^{-1}
Left atrium max volume	mL
Left atrium min volume	mL
Left atrium stroke volume	mL per beat
Left atrium ejection fraction	%
Right atrium max volume	mL
Right atrium min volume	mL
Right atrium stroke volume	mL per beat
Right atrium ejection fraction	%

Supplementary Table 4. Image-derived measures for benchmark data. We used the following MRI-derived clinical measurements in place of the spatiotemporal cardiac motion data to assess its discriminative power.

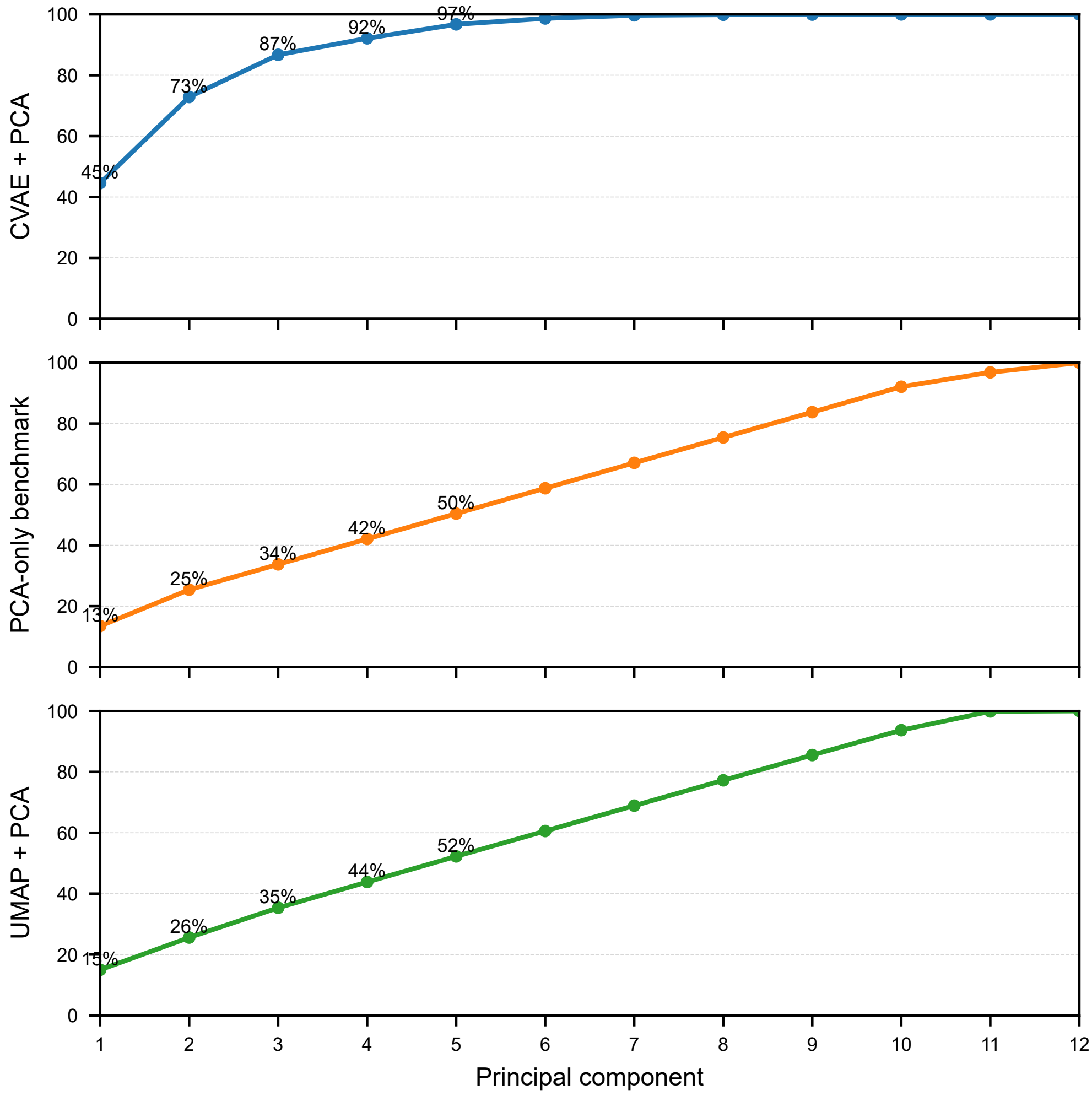

Supplementary Figure 6. Scree plot of explained variance. Cumulative explained variance as a function of the number of principal components for the CVAE+PCA pipeline (our model), the benchmark PCA-only model, and the UMAP+PCA benchmark. The CVAE+PCA pipeline achieves faster variance concentration, confirming the effectiveness of the non-linear encoding as a pre-processing step.

	Data	Model	Clustering	Stability (ARI)
Our model	High-dimensional meshes	CVAE+PCA Non-linear	Time series K-means	In-plane position: 0.91 Contour: 0.90
Benchmark data	Tabular MRI-derived measures	UMAP+PCA Non-linear	K-means	0.82
Benchmark model	High-dimensional meshes	PCA Linear	Time series K-means	In-plane position: 0.50 Contour: 0.85

Supplementary Table 5. Comparison of our model to two benchmarks. The benchmark against our data substituted cardiac point clouds with tabular MRI-derived data. The benchmark against our model used a simple principal component analysis (PCA) applied to the windows of cardiac motion. Our model achieved better cluster stability and robustness. ARI, average Rand index score; CVAE, convolutional variational autoencoder; UMAP, uniform manifold approximation and projection.

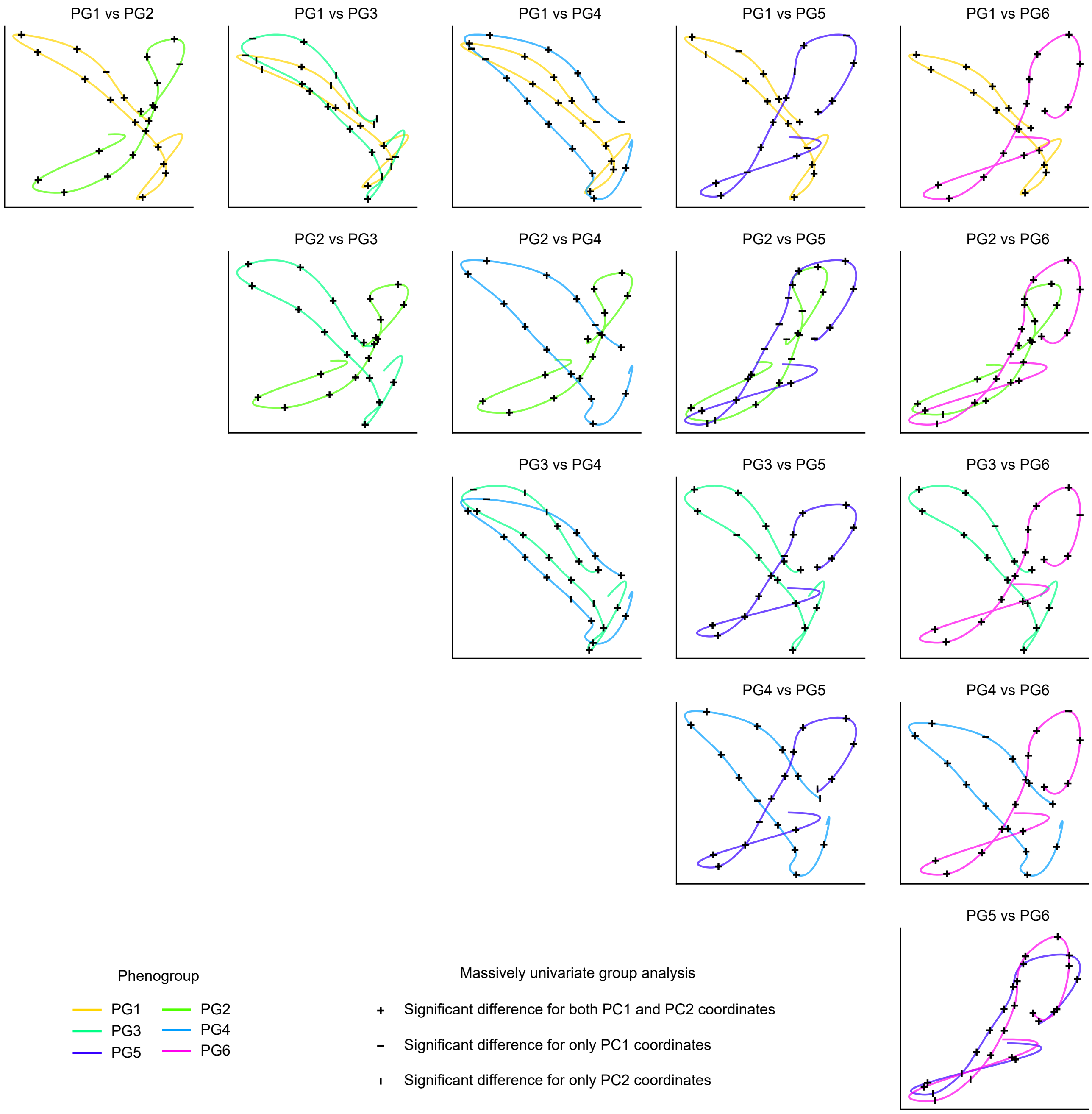

Supplementary Figure 7. Mass univariate group analysis of geometry differences of temporal signatures A mass univariate group analysis with permuted ordinary least squares (OLS) was performed to find geometry differences of the temporal signatures between phenogroups. The coordinates of the points making the trajectory in the space of the first and second principal components (PC1 and PC2) were compared between pairs of phenogroups. Benjamini-Hochberg correction was applied to account for multiple testing. The significance level was $P < 0.05$ and the sample size was n = 20,318. Comparisons were done for PC1 and PC2 separately. The average signatures of the phenogroups were plotted, and each trajectory point was annotated by a symbol indicating a significant difference between PC1, PC2, or both for the pair of phenogroups.

Phenotype	ICD 10	ICD 10 String	Phenocode
Atherosclerosis	I25.0	Atherosclerotic cardiovascular disease, so described	440
Atherosclerosis	I70	Atherosclerosis	440
Atherosclerosis	I70.8	Atherosclerosis of other arteries	440
Atherosclerosis	I70.9	Generalized and unspecified atherosclerosis	440
Cardiac arrest	I46	Cardiac arrest	427.42
Cardiac arrest	I46.0	Cardiac arrest with successful resuscitation	427.42
Chronic pulmonary heart disease	I27.1	Kyphoscoliotic heart disease	415.2
Chronic pulmonary heart disease	I27.8	Other specified pulmonary heart diseases	415.2
Chronic pulmonary heart disease	I27.9	Pulmonary heart disease, unspecified	415.2
Coronary atherosclerosis	I24.0	Coronary thrombosis not resulting in myocardial infarction	411.4
Coronary atherosclerosis	I25.1	Atherosclerotic heart disease	411.4
Dilated cardiomyopathy	I42.0	Dilated cardiomyopathy	425.1
Essential hypertension	I10	Essential (primary) hypertension	401.1
Heart failure NOS	I50	Heart failure	428.2
Heart failure NOS	I50.1	Left ventricular failure	428.2
Heart failure NOS	I50.9	Heart failure, unspecified	428.2
Hypertrophic cardiomyopathy	I42.1	Obstructive hypertrophic cardiomyopathy	425.11
Hypertrophic cardiomyopathy	I42.2	Other hypertrophic cardiomyopathy	425.12
Mitral valve disease	I05	Rheumatic mitral valve diseases	394.2
Mitral valve disease	I05.0	Mitral stenosis	394.2
Mitral valve disease	I05.1	Rheumatic mitral insufficiency	394.2
Mitral valve disease	I05.2	Mitral stenosis with insufficiency	394.2
Mitral valve disease	I05.8	Other mitral valve diseases	394.2
Mitral valve disease	I05.9	Mitral valve disease, unspecified	394.2
Mitral valve disease	I08.1	Disorders of both mitral and tricuspid valves	394.2
Mitral valve disease	I34.0	Mitral (valve) insufficiency	394.2
Mitral valve disease	I34.1	Mitral (valve) prolapse	394.2
Myocardial infarction	I21	Acute myocardial infarction	411.2
Myocardial infarction	I21.0	Acute transmural myocardial infarction of anterior wall	411.2
Myocardial infarction	I21.1	Acute transmural myocardial infarction of inferior wall	411.2
Myocardial infarction	I21.2	Acute transmural myocardial infarction of other sites	411.2
Myocardial infarction	I21.3	Acute transmural myocardial infarction of unspecified site	411.2
Myocardial infarction	I21.4	Acute subendocardial myocardial infarction	411.2
Myocardial infarction	I21.9	Acute myocardial infarction, unspecified	411.2
Myocardial infarction	I22	Subsequent myocardial infarction	411.2
Myocardial infarction	I22.0	Subsequent myocardial infarction of anterior wall	411.2
Myocardial infarction	I22.1	Subsequent myocardial infarction of inferior wall	411.2
Myocardial infarction	I22.8	Subsequent myocardial infarction of other sites	411.2
Myocardial infarction	I22.9	Subsequent myocardial infarction of unspecified site	411.2
Myocardial infarction	I23	Certain current complications following acute myocardial infarction	411.2
Myocardial infarction	I23.0	Haemopericardium as current complication following acute myocardial infarction	411.2
Myocardial infarction	I23.1	Atrial septal defect as current complication following acute myocardial infarction	411.2
Myocardial infarction	I23.2	Ventricular septal defect as current complication following acute myocardial infarction	411.2
Myocardial infarction	I23.3	Rupture of cardiac wall without haemopericardium as current complication following acute myocardial infarction	411.2
Myocardial infarction	I23.6	Thrombosis of atrium, auricular appendage, and ventricle as current complications following acute myocardial infarction	411.2
Myocardial infarction	I23.8	Other current complications following acute myocardial infarction	411.2
Myocardial infarction	I24.1	Dressler syndrome	411.2
Myocardial infarction	I25.2	Old myocardial infarction	411.2
Myocardial infarction	I51.0	Cardiac septal defect, acquired	411.2
Myocardial infarction	I51.3	Intracardiac thrombosis, not elsewhere classified	411.2
Other cardiomyopathy	I42	Cardiomyopathy	425.1
Other cardiomyopathy	I42.3	Endomyocardial (eosinophilic) disease	425.1
Other cardiomyopathy	I42.4	Endocardial fibroelastosis	425.1
Other cardiomyopathy	I42.5	Other restrictive cardiomyopathy	425.1
Other cardiomyopathy	I42.6	Alcoholic cardiomyopathy	425.8
Other cardiomyopathy	I42.7	Cardiomyopathy due to drugs and other external agents	425
Other forms of chronic heart disease	I51.1	Rupture of chordae tendineae, not elsewhere classified	414
Other forms of chronic heart disease	I51.2	Rupture of papillary muscle, not elsewhere classified	414
Other forms of chronic heart disease	I51.5	Myocardial degeneration	414
Other forms of chronic heart disease	I51.8	Other ill-defined heart diseases	414
Other forms of chronic heart disease	I51.9	Heart disease, unspecified	414
Other forms of chronic heart disease	I52	Other heart disorders in diseases classified elsewhere	414

Supplementary Table 6. Phenotypes and associated ICD-10 codes. Cardiovascular diseases and outcomes were grouped under related phenotypes for subsequent analysis.

References

1. Abraham A, Pedregosa F, Eickenberg M, Gervais P, Mueller A, Kossaifi J, Gramfort A, Thirion B & Varoquaux G. Machine learning for neuroimaging with scikit-learn. *Front Neuroinform* **8,** 14. doi:10.3389/fninf.2014.00014 (2014).
